# A new approach for improving access to primary care and public health in India: the 3T Model

**DOI:** 10.34172/hpp.025.44221

**Published:** 2025-12-30

**Authors:** Satadal Saha, Bibaswan Basu, Ruchira Mukherjee

**Affiliations:** ^1^Research and Development Unit, Foundation for Innovations in Health, Kolkata, India; ^2^School of Health, Environment and Sustainability Studies, TCG CREST (Deemed to be University), Kolkata, India

**Keywords:** Community health workers (CHWs), Electronic health record, Health services accessibility, Medically underserved areas, Primary health care, Task shifting

## Abstract

Health inequities in India encompass limited primary care access, dearth in healthcare personnel, especially in rural fragile geographies. A health system strengthening (HSS) approach through an innovative 3T-model can solve this ubiquitous problem. 3T includes- (i) Train rural youths (majorly women) as nationally certified & digitally literate Community Health Workers (CHWs) creating livelihood and women empowerment (ii) Technology: clinical algorithm driven software; easy-to-use affordable innovative diagnostic devices, screening tools (iii) Task-shifting: the software guides CHWs transmit structured, analysable, patient data to ‘remote’ doctors. CHWs addresses personnel shortage in health sector. Technology and task-shifting enable improved access to general and specialist doctors. Digital presence reduces doctors’ travel and time, enabling more consultations. The 3T-model addresses critical health inequities in India. Effective scaling can significantly improve access to affordable primary care and public health services for last-mile populations with potential to reduce gender disparities in access to essential healthcare.

## Graphical Abstract

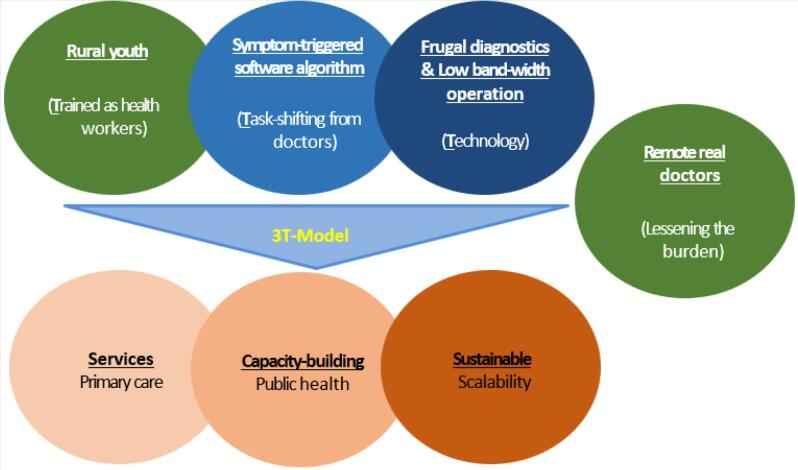

## Introduction

 Income and health inequity perpetuate poverty and ill-health. This fate is suffered by almost 50% of global population,^1^ concentrated in the global south. In India, the convergence of multiple factors has substantially compromised access to basic healthcare. These factors also limit access to public health programs, especially for the people living in remote rural and fragile geographies.^2^ The factors include –

- Shortage of doctors and health workers,
- Implementation and infrastructure challenges in the last mile areas,
- Technology deficit- the available technology is too expensive, too complex to operate in resource-poor environment.^3-8^

The resource poor settings of rural India is exposed to chronic inadequacy of appropriate healthcare access, making health inequities disproportionately higher relative to urban settings.^9^ The rural areas harbour majority of the Indian population (72%). Lack of access to affordable primary healthcare and public health among this 72% of the Indian population leads to increase in the societal disease burden. This also increases the vulnerability of the community to advanced stage of diseases. These factors cumulatively result in delayed presentation of illness and exposure to expensive, complex treatment. This culminates in health-related poverty shock and significant adverse outcomes.^10^

 Access to primary care in developing countries, including India, is influenced by gender according to previous research. Health seeking behaviour is markedly different between men and women, especially in rural settings. Due to inadequate access to appropriate primary care and public health, rural women are more susceptible to hospitalization with complexity than their male counterparts.^10,11^ Developing novel approaches is crucial for addressing the barriers to accessible and affordable healthcare in rural geographies; implement telehealth, mobile clinic specific models, emphasis on policy interventions and community health workers (CHWs).^2^

 CHWs and other Allied Health Professionals (AHPs) play a crucial role in healthcare access. India has a shortage of about 6.4 million AHPs,^12^ distributed across both hospital and community level. Unavailability of skilled AHPs significantly affects the quality of institutional and community healthcare limiting the growth of health sector.

 Previous studies fostering growth and development in the healthcare sector has recommended using technology for health management. In 2013, Praveen et al., documented the use of a mobile device-based clinical decision support system to manage blood pressure in individuals with higher risk of cardio-vascular disease.^13^ A relatively recent report, in 2022, documented the I-TREC model of care. Implemented in Punjab, India, I-TREC integrates clinical decision-support software (CDSS) and task-shifting for management of hypertension and diabetes through routine care.^14^ Another case study in South India, investigated if reorganization of work flow with task redistribution and follow-up of patients by CHWs improved the quality of healthcare delivery in primary care clinics. The authors reached the conclusion that while reorganization and follow-ups by CHWs are essential to delivering quality care, implementation requires capacity building in local primary care teams.^15^ While previous models (e.g., I-TREC, workflow reorganization) have addressed disease-specific challenges, there remains a gap in comprehensive, community-anchored, and scalable primary care models that integrate training, technology, and task-shifting. Despite being impactful, these initiatives also highlight the requirement for transitioning from fragmented, disease-specific or healthcare personnel-based models to comprehensive primary care models reinforced with permanent capacity building in the communities. The 3T model which integrates training for CHWs and task-shifting through use of technology can be a solution.

 Inadequate access to affordable primary care and public health for the rural population of India, accounting for approximately 72% of the total population, can be addressed through digital health program led by remote doctors and driven by trained, nationally certified, digitally literate, and technology-savvy CHWs. Creating a resource pool of rural health workers will address the significant shortage of AHPs, including CHWs. An estimated 6.4 million AHPs are needed to bridge the health sector workforce gap in India. Therefore, **T**raining rural youth, shifting skill-based **T**asks from doctors to CHWs, and leveraging deep science-driven, frugal **T**echnology can serve as an effective model - the 3T model - for addressing the shortage of AHPs, creating livelihood opportunities for the rural youth, and improving access to primary care and public health services, including mass population screening, for the rural underserved communities.

 Training women from the community and engaging them in the service delivery will contribute to women’s empowerment. Preventive and primary care programs driven by women CHWs have the potential to increase adoption rates among female community members, further strengthening the impact of the 3T model. This approach will help reduce gender-based disparities in access to healthcare for women.

 Resource constraints, inadequate infrastructure, complex health technologies, implementation challenges of public health policies across India’s vast population culminate in and exacerbates multidimensional poverty. Considering the disease burden in India, it is essential to develop and translate an integrated model to deliver primary care and public health for the last mile population. This perspective underlines the 3T model as the solution to filling the gap in access to healthcare among the underserved population. Training rural youth as CHWs, affordable technology and task-shifting from doctors to CHWs, when integrated, can improve access to primary care and public health besides generating employment to combat multidimensional poverty. This 3T model aims to address the twin burdens of health and income inequity in the society.

 This perspective article aims to present the 3T model as an integrated, scalable approach to strengthening primary healthcare and public health access in rural India. Specifically, it highlights how training, technology, and task-shifting can reduce inequities while simultaneously addressing income generation and women’s empowerment.

## Integrated approach to address health inequity in India

 Massive shortage of doctors, including their maldistribution between urban and rural areas, is not going to be solved in the short to mid-term (if ever). Focus on planning, development and implementation of a ‘Digital Model’ of delivery has been emphasized by the Government of India under National Digital Health Mission (Ayushman Bharat Digital Mission).^16^ This follows the success of digital technology adoption in India among the masses in other sectors such as financial sector and routine governance.

 Digital health model offers clear technological and clinical superiority over conventional telemedicine model. This model has following features -

- Evidence – based, actionable and analysable clinical information
- Reduced doctors’ consultation time; improved quality
- Comprehensive (consultation, tests, medicines, health products, public health)
- High replicability and scalability (low band-width, language/location-agnostic, less doctors)
- Dynamic and versatile – eco-system approach for public health programs

Capacity building among marginalized and disenfranchised section of the society as health workers will significantly improve income of those families through sustainable employment in the health sector (in wage and self-employment). What remains unique in AHP training and placement programs is preferential employment of women thus contributing to women’s empowerment. Healthcare is and will remain the leading employment provider in India.^12^

 Primary care constitutes the overwhelming ‘need;’ and this has got to be solved first – across infectious, non-communicable and deficiency diseases. According to the World Health Organization, by the year 2030, 60 million lives could be saved through scaling up primary health care interventions across low- and middle-income countries and increase average life expectancy by 3.7 years.^17^ In primary care, adequate treatment through a good history and basic diagnostic tests and establishment of meaningful communication between the CHWs at front-end and the ‘real’ doctor at the back-end save the doctors’ time; eliminates travel and inconvenience. Balanced availability of all three tiers of care, including health education, ensures good health for the individual, society, and nation. But no progress can be made if the issue of primary care remains unresolved while we continue to make progress across the secondary and tertiary segments by setting up more hospitals.

## 3T model – Implementation Framework

**Figure 1:**
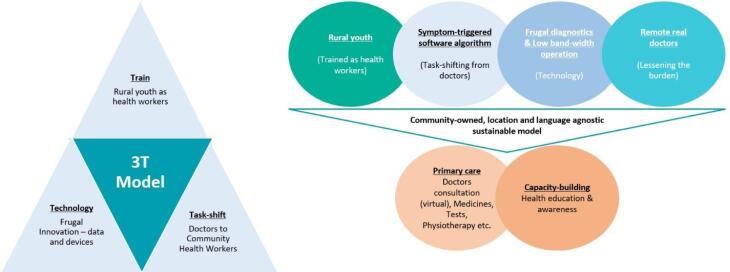
The 3T-Model for Improving Access to Primary Care and Public Health

 Through engagement of stakeholders and experiential deep learning, an innovative and integrated 3T model (**T**raining, **T**ask-shifting and **T**echnology) has been developed and implemented to address the health inequity in India (Figure 1).

###  Training of Rural Youth as CHWs

 The Local Youth (N = 174, 61% Females), Predominantly from Scheduled Caste and Scheduled Tribe Background Were Enrolled to Pursue Training as Frontline Health Worker. The Minimum Qualification Required Was Class X. The Selection Process Was Undertaken in Line with The Guidelines Laid Down by The Affiliation Body and West Bengal Scheduled Castes, Scheduled Tribes and Other Backward Classes Development and Finance Corporation (A Government of West Bengal Undertaking).

###  Training Specifics

 Local Youth Were Trained as Frontline Health Worker Which Is a National Skills Qualifications Framework (NSQF) Aligned Curriculum (Qualification Pack National Occupational Standard – HSS/Q8601). After Successful Completion of The Course, The Youth Received Certificates from Healthcare Sector Skill Council Under National Skill Development Corporation, Ministry of Skill Development & Entrepreneurship, Govt. Of India. The Curriculum Was Enhanced by Inclusion of Digital Rural Primary Care and Public Health-Oriented Pedagogy – Digital Literacy, Use of Diagnostic Devices, Clinical Communication, Medicine Management & Prescription Reading, History Taking, Physical Examination, Software, Financial Literacy, Dental and Mental Healthcare. Upskilling Program for The CHWs Is Continuous.

###  Task-Shifting and Technology Dissemination

**Figure 2:**
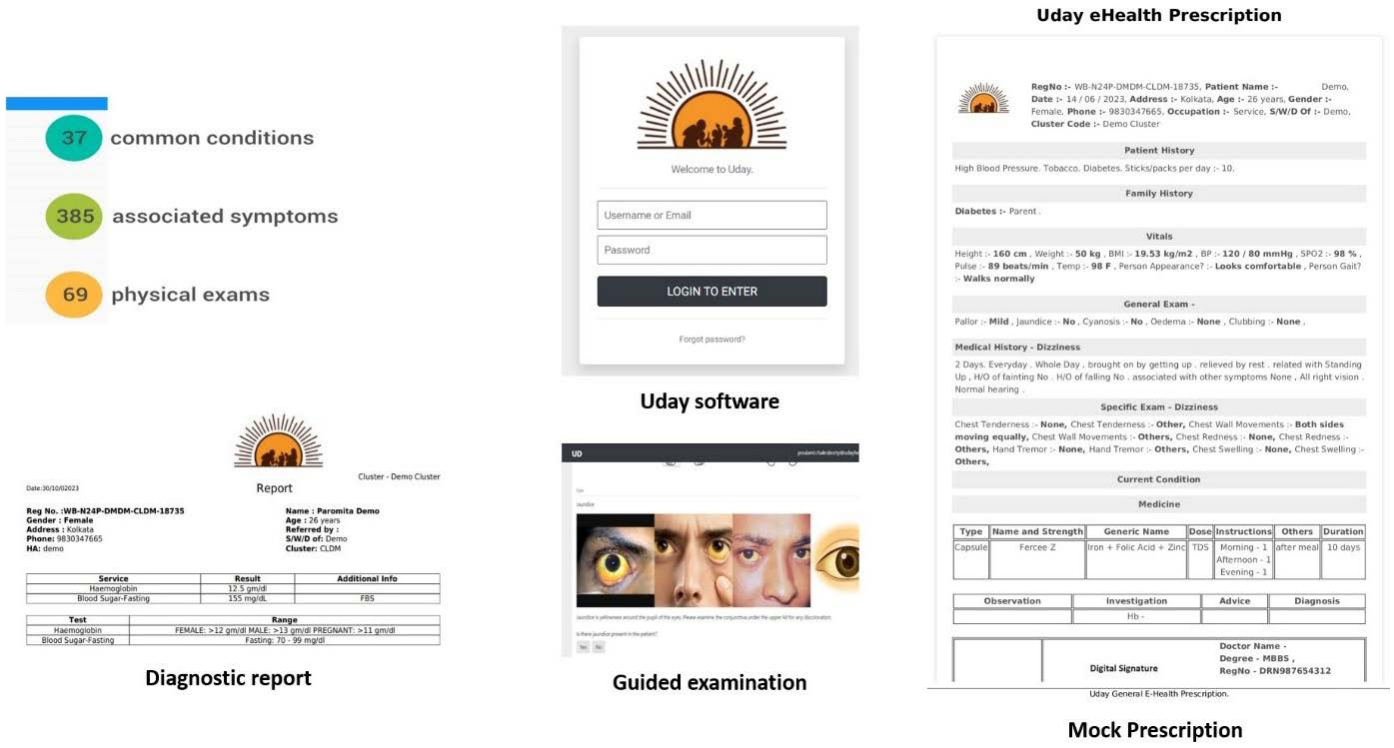
The Clinical Decision Support Software (CDSS)

 User Friendly CDSS (Designed with The Users) (Figure 2) And an Array of Technologies Was Developed. These Technologies Are Simple to Use, Affordable, Accurate and Reliably Perform in The Constrained Rural Environment. These Technologies Help Shift a Significant Part of The Doctors’ Tasks from The Doctor to The Front-End CHWs. Reliable, Structured, And Analysable Health Data from The Patients, Which Form the Basis of Decision-Making by The Doctors, Are Transferred Real-Time for the ‘Remotely’ Located Doctors to Make Appropriate Clinical Decisions.

###  Delivery of Services

**Figure 3:**
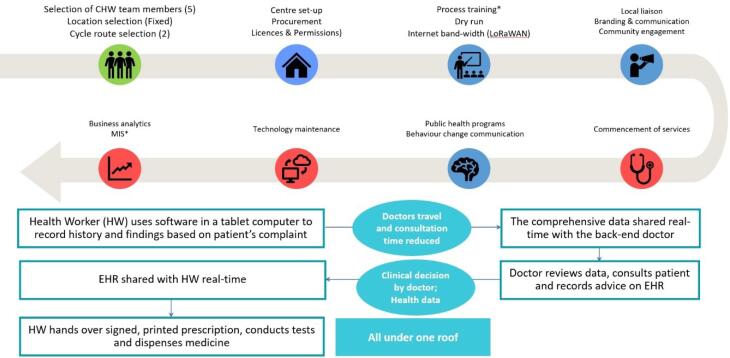
Operational Procedure of The Digital Clinics

 Trained And Certified Digitally Literate CHWs Use the Software on Their Tablet Computers to Obtain History from The Patient, Based on The Patient’s Medical Complaint, Using Its Symptom-Triggered Algorithm. They Also Undertake Basic Physical Examination of The Patient and Share the Health Data in An Electronic Health Record Format in Real-Time with Back-End Doctors (General Practitioner/Specialist). The Doctor Guides the Treatment Plan, Including Medicines, Diagnostic Tests, Physiotherapy, Dietary Advices Etc. Which Are All Provided by The CHWs During the Same Patient Visit. Figure 3 Shows the Operations of Digital Clinics.

 All the technologies are location and language agnostic. Blockchain based Long Range Wireless Area Network (LoRaWAN) technology can enable data transmission and security even in ultra-low/no band-width areas. The model has been implemented in collaboration with some of the ‘Institutes of National Importance’ and ‘Higher Educational Institutions’.

 This leads to dual reduction - the need for doctors to travel and live in rural locations, and their time per patient (by almost 70%), without reducing accuracy and safety of clinical decisions.

## Rural Digital Clinic Clusters (RDCC)

**Figure 4:**
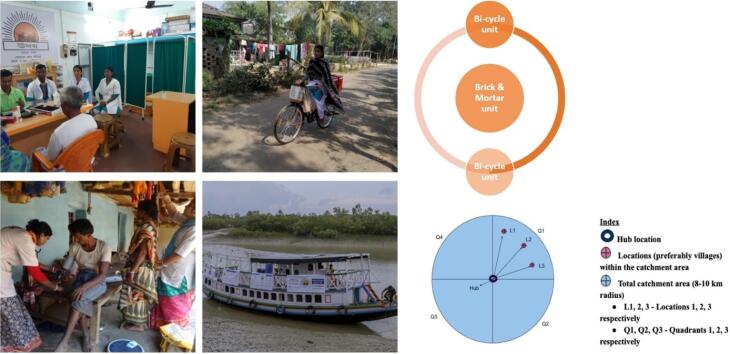
Digital Clinic Cluster Layout

 About 3,00,000 rural population were covered through the 40 digital clinics across various locations of West Bengal, India. The clinics were built in the form of a cluster, each cluster comprising – 1 fixed clinic (brick and mortar unit) in a village and 2 cycle borne units, each covering a radius of 10 kms around the fixed clinic in different directions (Figure 4). Each clinic employed 4 certified CHWs. A purpose-modified motor-boat at the Sundarbans (world’s largest delta and mangrove forest) covers 6 remote islands of West Bengal, India. Each cluster reaches between 20,000 and 25,000 rural residents. Services available in the RDCC include digital and in-person consultation (GP and specialist), vital signs checking, history taking and basic physical examination, diagnostic tests, medicines, physiotherapy demonstration, sanitary napkins, ORS etc. Dental health services are made available through well-equipped mobile dental van. Other services include mental health program, cancer risk assessment & screening, screening program for diabetes, hypertension and heart diseases, community health education and awareness, behaviour-change communication, School Health Program, WASH, National Program implementation.

 Between January 2022 and February 2025, the 3T model ensured preventive and primary care services to a total of 27,566 rural community members. A remarkable adoption rate of 60% was reached through the model for cancer screening (cancer of cervix, breast, and oral cavity) among women aged between 25 years and 65 years (n = 5009). This program was conducted between August 2024 and July 2025. The number of women screened is very low compared to the Indian population and the adoption rate must be interpreted with caution. The screening uptake for breast and cervical cancer exceeds the national averages of 0.9% and 1.9%, respectively.^18^ This contributed towards bridging a critical gap in women’s health. The adoption rate was observed to be 95% for diabetes, hypertension, anaemia among individuals aged 30 years and above. A study in 2019, reported the prevalence of hypertension screening to be 64% among 394 participants residing in Puducherry, India.^19^ Moreover, 23 school health programs were conducted reaching 2,422 students as an endeavour to foster health awareness at an early age. The above-mentioned data consist of anonymized, non-identifiable, aggregated service delivery records collected as part of the routine activities of RDCC and its extended network.

 In addition, RDCC were supported by two economy diagnostic centres, where more complex diagnostic tests that could not be conducted at the RDCC were performed. The economy diagnostic centres were supported by Common Research & Technology Development Hub on Affordable Healthcare at Indian Institute of Technology Kharagpur and a Municipal Corporation in West Bengal.

## Implications of the 3T model

 This model achieves 5 ‘A’ s of health system –

- Affordability - Increases household income and reduces spend on healthcare
- Availability - Abundantly available services enhance participation
- Accessibility - Easy accessibility for community members residing in resource poor settings
- Accommodation - Takes care of the needs of a large spectrum of society
- Acceptability - Community member-led; breaks down cultural & language barriers and gender disparity in health seeking behaviour; easier diffusion; rural community adoption of digital health services has been increased manyfold

The model also addresses the gender disparities in availing healthcare. 65% of the patients visiting the RDCC were women, which is higher than footfall in hospitals. A recent study conducted in All India Institute of Medical Sciences (AIIMS), New Delhi showed that of all the outpatients visiting the public-funded tertiary care hospital, only 37% were women.^20^ Closer proximity of RDCCs can not only reduce the travel time but also the associated cost of care. Lower out of pocket expenditure (OOPE) and the culturally congruent design of the RDCC service delivery might be an added factor contributing to comfort among women seeking care. This represents that when healthcare services are delivered closer to home and made more affordable, women avail more healthcare.

 This model is ‘fit-for-purpose’ solution for global application –

- Train rural youth as CHWs
- Task-shifting (doctors to CHWs) is the key element
- Customisable, easy-to-use frugal location-agnostic technologies
- Structured health data – Research & Development in public health, disease biology
- Software as a Service (SaaS)/Operation & Management
- Disruptive reduction of healthcare cost

This model has led to employment creation among rural youth and increase in rural household earning. The model advocates women’s empowerment as over 60% health workers are women, prevents urban migration and maintains family and social eco-system. Introduction of modern deep-science technologies into the community through the 3T model occurs in a culturally congruent manner, provides comprehensive primary care & public health services, including mental health, homecare for pregnant women, children, and elderly all under the same platform. This enhances access and reduces out-of-pocket expenses significantly. A recent study conducted in the state of Chhattisgarh, India, reported the average OOPE per episode of availing outpatient care. The assessment showed an OOPE of INR 400, 586 and 2643 respectively for public healthcare providers, informal private providers and formal for-profit providers respectively.^21^ The OOPE in the 3T model amounts to an average of INR 250 inclusive of doctor consultation, tests, and medicines.

## Risk management approach

 The risk mitigation approach has been tabulated in Table 1.

**Table 1:** Risk Management Approach

**Risks**	**Mitigations**
Multi-tasking ability of CHWs serving in the digital clinics	Frequent upskilling, Entrepreneurship development, Performance incentives
Technology sustenance in resource-poor areas, including internet band-width	Frugal innovation strategy, with deep understanding of design ethnography, LoRaWAN
Community acceptance	Behaviour-change communication, Diffusion of Innovation approach, Information, Education & Communication (IEC) material, Success stories, Community engagement strategies
Engagement of doctors	Lesson from COVID, Support from national policy on digital health, reduction of need to travel to remote places, less time/patient

## SWOT analysis of the 3T model

 A detailed Strength, Weakness, Opportunities and Threat (SWOT) analysis has been performed on the 3T model and tabulated below (Table 2).

**Table 2:** SWOT Analysis Of The 3T Model

**Strengths**	**Weaknesses**
Integrated approach creates a holistic system to address gaps in primary healthcare Scalable and replicable besides being location and language agonistic Livelihood creation among rural youth, providing sustainable rural income can reduce urban migration CHWs are majorly women, the model advocates women empowerment Permanent capacity in the community (CHWs), with transfer of agency Makes primary healthcare more accessible and affordable reducing the OOPE Technology helps build a reservoir of indigenous health data useful for research and policy design Technology guided data collection and patient handling reduces errors by CHWs Frugal technology ensures usability even in difficult geographies Reduces the doctor’s need to travel and saves times enabling doctors to consult more patients remotely The model is culturally congruent and community-led that can improve trust and service adoption rate Process-driven operation, with high level of quality assurance and error prevention Alignment with global health and development agendas such as Sustainable Development Goals (SDGs)- No poverty - > 1, Good health - > 3, Quality education - > 4, Gender Equality - > 5, Good jobs and economic growth - > 8, Reduced inequalities - > 10	Technology dependence in the fragile remote geographies can hamper sustained performance due to power supply issues CHWs need continuous training and upskilling Initial cost of setting up digital clinics and maintenance is capital intensive especially for scaling Sometimes, doctors may resist task-shifting to CHWs There is a risk of fragmentation, inefficiencies, and service duplication if the model is not well integrated with national or state health system Policy and regulatory barriers can slow adoption
Opportunities	Threats
Potential for global application Integration with National and global primary care and public health programs Advancements in technologies can be easily included in the model Technology can assist in dynamic monitoring of public health Public-private partnerships and collaboration with government / non-governmental organisations Structured health data can help manage outbreaks / epidemics better Early detection and management of diseases Behaviour changes communication and long-term change in health seeking behaviour	Rapidly evolving technology makes continuous upgrades essential Long-term viability depends on policy support; policy changes could impact services Barrier to adoption of digital health model persists in some rural communities Attrition among the CHWs- migration of trained CHWs to urban areas can undermine rural service continuity Need for strong cybersecurity of indigenous health data Competition with older / traditional healthcare models Climate catastrophe especially in fragile geographies Device sensor malfunction

 The 3T model is a strong, scalable, replicable model with potential to transform the healthcare landscape in India and beyond. Proper integration of health policies and continuous upgradation of technology, the model can become a benchmark for primary healthcare delivery.

## Future Research & Development (R&D) perspectives

 A significant amount of structured health data will be generated in an analysable format that can be a rich source of R&D in technologies, disease biology, and public health program design. Health data repository can not only enable evidence-based practice, but also endeavour to make personalized / precision medicine a reality. The repository can also be used for developing risk stratification algorithms to predict disease risk. Besides forecasting, prognosis of the disease based on medical history can also be determined. The same digital model can be further developed through device integration, machine learning algorithms, blockchain technology and integration of genomic, proteomic, metabolomic data into creation of heat maps & risk scores of various diseases. AI-driven risk stratification can be useful for better clinical decision making. AI-enabled software can also forecast expected higher occurrence of certain diseases based other confounding factors such as climate development. This can also be used for measurement of effectiveness of public health programs. This will enable better preparedness of public health systems, resource mobilisation, and targeted intervention.

## Conclusion

 With vast swathes of people living without access to primary care in the South-South geographies, and the health sector remaining a major driver of formal sector employment for skilled health workers, this integrated 3T model offers a compelling opportunity at scale for significant expansion in a language and location-agnostic manner with sustainability. Robust primary health care systems provide the most cost-effective foundation for low and middle income countries (LMICs) to achieve universal health coverage (UHC), build resilience against health shocks, and contribute to the overall population health and well-being.^22^ According to the World Health Organization (WHO), there is a projected shortfall of 11 million health workers by 2030, predominantly across LMICs.^23^ Given the similar challenges faced by LMICs - including health workforce shortages and inadequate access to affordable healthcare – this culturally congruent model can be effectively adapted and replicated in contexts of other LMICs.^24^

 The 3T model requires minimal infrastructure, continuous upskilling of health workers. This involves innovative health technology dissemination which can be achieved through collaboration with science & technology institutions. This model can be easily integrated with the government health system to ensure appropriate and timely referral. Scaling the 3T model requires policies recognizing task-shifting, wide adoption of digital health program, and frugal technology innovation, availability of skilled CHWs. The model aligns very well with the global mandates of promoting digital technologies in delivery of healthcare. The 3T model can be easily adapted for any country by aligning with the country’s national health priorities, healthcare regulations and existing CHW systems. Engaging ministries and NGOs as stakeholders can ensure policy fit. By design, the 3T model complements the National Digital Health Mission, known as the Ayushman Bharat Digital Mission - through its focus on skilled workforce, data-driven approach and technology enabled service delivery. Under the National Qualification Register (NQR) of the Government of India, modules related to telehealth, health promotion, digital literacy, communication skills, entrepreneurial skills, history taking, physical examination etc. are available as part of specific job roles. A comprehensive, NSQF-aligned curriculum on digital health, focusing on the 3T model, can be developed and implemented at scale to train rural youth across the country.

 The model can easily be incorporated with programs to mitigate diseases with high burden. The training of the health workers can be tailored to the local culture, language, and qualification framework. The 3T model aims to provide data-driven, evidence-based, affordable primary care and public health services to last-mile populations, encompassing health promotion, disease prevention, basic treatment, and referral for advanced care - aligning with the goals of UHC. This model has positive impact on multiple Sustainable Development Goals (SDGs) –

 SDG 1 – No Poverty: by generating livelihoods and reducing the cost of care;

 SDG 3 – Good Health and Well-Being: by improving access to primary care and public health services for the community members;

 SDG 4 – Quality Education: by providing high-quality training to rural youth;

 SDG 5 – Gender Equality: building capacity among large numbers of women from the community and expanding access to essential healthcare services for female community members;

 SDG 8 – Decent Work and Economic Growth: by creating in-wage employment opportunities for the rural youth in the health sector;

 SDG 10 – Reduced Inequalities: by educating rural youth as CHWs, creating livelihood and permanent capacity in the community, delivering accessible preventive and primary care services for the disenfranchised section of the society.

 This model enables the vision of “Health for a Billion.”

## Competing Interests

 This is to declare that the authors do not have any competing financial and personal interest.

## Ethical Approval

 Not applicable.
